# Associations between Parents’ Digital Media Habits, Engagement, Awareness, and Movement Guidelines among Preschool-Age Children: International Ipreschooler Surveillance Study

**DOI:** 10.3390/ijerph191710484

**Published:** 2022-08-23

**Authors:** Hongzhi Guo, Jiameng Ma, Terence Buan Kiong Chua, Lee Yong Tay, Michael Yong Hwa Chia, Hyunshik Kim

**Affiliations:** 1 Graduate School of Human Sciences, Waseda University, Tokorozawa 169-8050, Japan; 2 Faculty of Sports Science, Sendai University, Shibata 989-1693, Japan; 3 National Institute of Education, Nanyang Technological University, Singapore 637616, Singapore

**Keywords:** preschool children, digital media variables, physical activity, screen time, sleep duration, 24-h movement guidelines, IISSAAR

## Abstract

The 24-hour movement guidelines (24-h MG) recommend behaviors (physical activity, screen time, sleep) to aid appropriate physical and mental development in early childhood. This research examined parents’ digital media habits (DMH), engagement (DME), and awareness (DMA) among parents in relation to their preschool-aged children’s 24-h MG in Japan and identified and compared the modifiable determinants of adherence to 24-h MG in urban and rural regions. This cross-sectional study included 867 participants and data were obtained from the International Ipreschooler Surveillance Study Among Asians and OtheRs (IISSAAR). The results revealed that adherence to weekend screen time recommendations and weekday sleep duration were higher in the urban region. The parents’ digital media variables that predicted moderate-intensity to vigorous-intensity physical activity among preschool-aged children were parents’ DME and DMA in the urban regions and parents’ DME in the rural regions. The children’s screen time was significantly associated with parents’ DMH, DME, and DMA in the urban regions and with parents’ DMH and DMA in the rural regions (*p* < 0.005, *p* < 0.001, respectively). This study confirmed that parents’ DMH, DME, and DMA are strong predictors of adherence to 24-h MG among preschool-aged children living in both rural and urban regions in Japan.

## 1. Introduction

Early childhood is an important period for physical and mental growth, during which children adapt to their environment, and this period is reported to have an impact on health behaviors in adulthood [1]. Therefore, during early childhood, promoting regular and adequate physical activity (PA), limiting screen time—defined as time spent watching TV or playing games—as much as possible, and ensuring adequate sleep serve as the basis for establishing a healthy lifestyle [2]. Each of the behaviors (PA, screen time, and sleep duration) recommended in the 24-h MG is repeated daily and routinized and thus can possibly be established as a lifestyle habit in preschool children. It is important to consider all three movement behaviors during a given 24-hour period for health [3]. The 24-hour movement guidelines (24-h MG) are an approach that transitions from single movement behaviors to integrated movement behaviors during the day and offers a new paradigm for thinking about movement behaviors. The three movement behaviors, PA, screen time, and sleep were developed in Canada [4] and disseminated by the World Health Organization (WHO) [5]. Furthermore, the 24-h MG (e.g., high PA, low screen time, adequate sleep) contribute to preschool children’s physical, psychological, and mental health as they develop a stronger immune system [3,6,7]. Nevertheless, many children across the globe do not engage in adequate amounts of moderate-intensity to vigorous-intensity PA (MVPA) [8,9], spend much of their time on screen-viewing [10,11], and sleep late at night or for short durations [12,13]. Such behaviors have been noted to have an adverse impact on the physical and mental health of children [14,15,16]. For these reasons, strategies to boost adherence to the 24-h MG among children are imperative and useful.

With the extensive access to digital media (DM) in recent years, the age at first screen exposure is progressively decreasing [17]. While DM does have benefits for preschool-aged children, such as educational benefits, informational access, social connection, and social support, excessive recreational screen time can have a detrimental impact on health and thus requires appropriate parental mitigation and guidance [18]. Parents’ use of DM is reported to alleviate parenting stress to some extent [19]; however, excessive screen use can negatively affect the health and lifestyles of users [20]. Hence, it is also necessary to investigate whether parents’ attitudes toward the use of DM influence their children’s screen time. Previous studies have revealed that parental participation, practices, and control of the home environment rather than perceived neighborhood factors were important determinants of successful interventions to reduce overweight and obesity in preschool children, and statistically significant changes and consistent association with screen time in children were also found [21,22,23]. Thus, 24-h MG of preschool children could be highly influenced by parents. Therefore, modifying behaviors recommended in the 24-h MG require an examination of parents’ DM habits, participation, and cognitive factors. Unfortunately, such studies are lacking. Furthermore, parenting participation differs considerably depending on the living environment, lifestyle, and socioeconomic factors between urban and rural dwelling households; therefore, it is also important to examine parents’ DM factors by rural and urban regions [24,25,26].

This study examined parents’ digital media habits (DMH), engagement (DME), and awareness (DMA) in relation to their preschool-aged children’s 24-h MG and identified the modifiable determinants of adherence to 24-h MG among preschool children living in urban and rural regions of Japan. The research questions are as follows: (i) are there regional differences in children’s 24-h MG? (ii) Does the association between parental’s DM factors and children’s 24-h MG differ across regions?

## 2. Materials and Methods

### 2.1. Study Design and Participants

This is a cross-sectional study, and data were obtained from the International Ipreschooler Surveillance Study Among Asians and OtheRs (IISSAAR) [24,27]. Using the classifications of city, town, and village from the 1947 Japanese Enforcement Decree of Statutes of Local Governments, which were based on (i) population, (ii) number of households, (iii) occupation, and (iv) city facilities, Nishinomiya was chosen for the urban region (population: 487,800, area: 99.96 km²) and Ōhira-mura (population: 5918, area: 60.32 km²) and Tomiya-shi (population: 52,430, area: 49.18 km²) constituted the rural region [25]. In addition, 3 of the 51 nursery schools in Nishinomiya that consented to participate in our study were selected as the urban subjects and 5 of the 9 nursery schools in Ōhira and Tomiya were selected as the rural subjects. Three childcare facilities in Nishinomiya (urban) and five childcare facilities in Ōhira and Tomiya (rural) that completed a questionnaire survey between June and October 2019 were selected via convenience sampling (Japanese childcare center age of use: 0–6 year old). Appropriate assent and consent were obtained from parents and teachers in accordance with the declaration of Helsinki, and data from 867 participants who signed the informed consent form (52.7% boys; 47.3% girls, 67.1% retrieval rate) were analyzed. The study received prior approval from the Sendai University Ethics Committee, Faculty of Sports Science, Japan (SU2019-31), and the equivalent of that from Nanyang Technological University, Singapore (IRB 2019-02-036).

### 2.2. Measures

#### 2.2.1. Parents’ Digital Media Factors

The SMALLQ^®^ developed by Chia et al. was used [27] after translating the items into Japanese and discussing with the original authors of SMALLQ^®^ as per the WHO process of cultural adaptation [28]. In the present study, the internal consistency (Cronbach’s alpha) of the SMALLQ^®^ was established as 0.71: an acceptable level [29] based upon parent-reported self and child digital media use on the weekday and weekend. The SMALLQ^®^ consisted of 25 questions, including questions on digital media habits of the child and the parent (Segment I), nondigital media behavior of the child (Segment II), and background information on the parent and child (Segment III). To achieve our study purpose, of the 25 items, we extracted only those related to parents and redefined them as follows:

DMH: parents were asked about their digital habits on a typical weekday and weekend. The choices were segregated as used for (i) entertainment and (ii) social networking.

DME: regarding parental co-participation in children’s digital media and physical play, parents were asked to estimate their percent engagement in their child’s total time on weekdays and weekends. An example was given: (i) when your child uses media, estimate the amount of time you are engaged with him/her (e.g., interacting with your child while watching videos together); (ii) estimate the percentage of time you engage with your child in indoor and outdoor physical activity (e.g., playing hide-and-seek together).

DMA: parents were asked to indicate whether they are aware of the three professional guidelines on digital media use by children and if they practiced those guidelines. The guidelines are (i) limit digital media use for children younger than two years, (ii) limit screen time to 1 h per day for children 2–5 years, (iii) introduce only high-quality educational programs for children 18–24 months.

#### 2.2.2. Physical Activity

PA was assessed based on two free-response items on the SMALLQ^®^: (i) indoor play (e.g., dancing, crawling, playing with concrete manipulative toys); (ii) outdoor physical play (e.g., playing hide-and-seek in a playground). PA was calculated by: Indoor play (hrs) + Outdoor play (hrs) = Total physical activity (hrs). Moderate-to-vigorous physical activity (MVPA) was calculated by: Total physical activity (TPA) × average % of MVPA = MVPA (hrs) [27].

#### 2.2.3. Screen Time

Screen time was assessed based on two free-response items about leisure time on the SMALLQ^®^, and weekdays and weekends were surveyed separately: (i) using media for entertainment (e.g., watching shows, playing games, listening to music); (ii) communicating (e.g., chatting with relatives via Facetime/Skype) [27].

#### 2.2.4. Sleep Duration

Sleep duration was measured using the questions: (i) how many hours does your child sleep at night?; (ii) how long is your child’s nap time? Daily sleep duration was calculated by weekdays and weekends as follows: ((night sleep time + nap time)/2) [16].

#### 2.2.5. 24-hour Movement Guidelines

The following recommendations were used to evaluate the new 24-h MG for preschool children: PA guidelines, 180 min of total PA including 60 min/day of moderate to vigorous PA; screen time guidelines, less than 1 h per day; and sleep duration guidelines, 10–13 h within 24 h [5,30].

### 2.3. Demographic Factors

Demographic factors included sex, age, height, weight, and BMI z-score for preschool children [31] and parents’ sex, age, height, weight, and BMI. The data were collected from the parents.

### 2.4. Statistical Analysis

Data from 867 Japanese preschool children (urban region: 489, rural region: 378) who provided complete information on the study variables were analyzed using three models. First, frequency analyses were conducted to investigate the percentage of preschool children who met the PA guidelines, screen time guidelines, and sleep guidelines or combinations of these guidelines. Descriptive statistics were used to investigate the total duration of MVPA, screen time, and sleep duration for 1 week, including weekdays and weekends, and continuous variables were presented as means and standard deviations. Second, independent *t*-tests were performed to analyze the differences in parents’ DMH, DME, and DMA between urban and rural regions. We used *t*-tests to examine changes in adherence to the 24-h MG (i.e., PA, screen time, and sleep duration) between urban and rural samples. Third, PA, screen time, and sleep duration were analyzed using a univariate linear regression analysis to analyze the association with each of the 24-h MG parameters in the urban and rural regions. In addition, a multivariate model adjusted for sex, age, and BMI was also used. All variables included in each of the multivariate models were assessed for multicollinearity, which is prevalent among parents’ DMH, DME, and DMA [32]. All statistical analyses were conducted using IBM SPSS 26.0 (IBM, Armonk, NY, USA), and the level of significance was set at *p* < 0.05.

## 3. Results

### 3.1. Study Population

Table 1 compares children’s and parents’ characteristics between regions. Among children, 52.7% were boys, and 47.3% were girls. The proportion of girls was slightly higher in the urban region. Age was higher in the urban regions (4.6 ± 0.9 years) than in the rural regions (4.5 ± 0.9 years). Body weight and BMI were significantly higher in the rural than in the urban regions (*p* < 0.001, *p* < 0.034, respectively). Among parents, the proportion of mothers was higher in the urban regions (93.9%) than in the rural regions (89.2%) (*p* < 0.017). Parental age and adult obesity were also higher in the urban regions compared to the rural regions (both, *p* < 0.001).

### 3.2. Parents’ Digital Media Variables of Urban and Rural

Table 2 shows the mean and standard deviation of DM variables and the regional gap among parents. Regarding parents’ DMH, parents in the rural regions spent a significantly longer time on DM for “Weekend entertainment” and for “Total entertainment for the week” (*p* < 0.001, *p* < 0.016, respectively). Regarding parents’ DME, parents in rural regions showed significantly longer “Digital media engagement with their children (weekend)” (*p* < 0.041). Regarding parents’ DMA, parents in the urban region showed significantly higher awareness regarding “Limiting screen time to 1 h per day for children 2–5 years” and “Introducing only high-quality educational programs for children 18–24 months” (*p* < 0.038, *p* < 0.030, respectively) (Table 2).

### 3.3. Urban and Rural Differences in Children’s 24-h MG

Table 3 shows the differences in PA, screen time, and sleep duration between children living in the urban and rural regions. The mean weekday MVPA was higher among urban children (26.8 ± 31.9 min) than rural children (20.4 ± 38.3 min). However, weekend screen time was higher among rural children (143.7 ± 95.9 min) than urban children (113.6 ± 80.2 min). Regarding adherence to 24-h MG, adherence rates to screen time on the weekend (*p* < 0.005), and sleep duration during weekdays (*p* < 0.001), these were higher among urban children than among rural children (Table 3).

### 3.4. Parents’ Digital Media Variables Associated with 24-h MG in the Urban Regions

In the multivariate regression analysis, after adjusting for sex, age, and BMI to analyze the relationship between adherence to 24-h MG and parents’ DM variables, MVPA was positively associated with “Physical play engagement with children,” “Digital media engagement with child,” “Limit digital media use for children younger than 2 years,” and “Limit screen time to 1 h per day for children 2–5 years.” Further, screen time was positively associated with “Entertainment” and negatively associated with “Physical play engagement with children,” “Limit digital media use for children younger than 2 years,” and “Limit screen time to 1 h per day for children 2–5 years” (Table 4).

### 3.5. Parents’ Digital Media Variables Associated with 24-h MG in the Rural Regions

**Figure 1:**
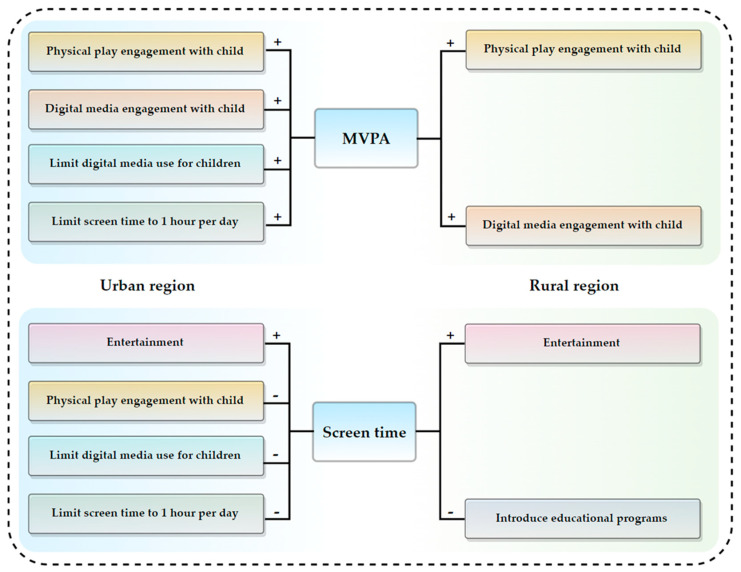
Proposed the relationships between urban/rural parents’ digital media habits, engagement, awareness, and 24-h MG for preschool children.

The same statistical methods were used to analyze the associations between parents’ DM variables with 24-h MG in rural regions. Children’s MVPA was positively associated with “Physical play engagement with child” and “Digital media engagement with child”. Screen time was positively associated with “Entertainment” and negatively associated with “Introducing only high-quality educational programs for children 18–24 months” (Table 5 and Figure 1).

## 4. Discussion

This research is part of a multinational study called the International Ipreschooler Surveillance Study Among Asians and OtheRs (IISSAAR) that analyzed the parents’ DMH, DME, and DMA in relation to preschool children’s 24-h MG in Japan by region (urban and rural).

First, 24-h MG (MVPA, screen time, sleep duration) of preschool children living in the urban and rural regions in Japan were compared. Weekday MVPA was longer in the urban regions, while screen time on the weekends was longer in the rural regions. Regarding adherence to 24-h MG, adherence to weekend screen time recommendations was higher in the urban regions, and weekday sleep duration was also higher in the urban regions. Although the correlation between the area of residence (regional gap) and movement behavior must be examined to gain a deeper understanding of movement behavior patterns among preschool-aged children [33], relevant research on preschool-aged children is relatively lacking compared to research in older age groups of children. In a Mozambique study on elementary school students, rural children demonstrated higher adherence to MVPA, sleep duration, and screen time recommendations [34]. In a study conducted in China, which has a similar culture and lifestyle to that of Korea, elementary school students in urban regions showed higher adherence to MVPA and screen time recommendations, while those in rural regions showed high compliance with sleep duration recommendations [35]. The higher levels of MVPA among urban children in Japan may be attributed to the presumably easier access to community sports programs or sports facilities [36] and higher economic standards of the urban regions; where in such contexts, families with a higher socioeconomic status would be more aware of the locations of recreational facilities and can afford or better utilize the equipment for exercise [35]. In another Asian study, rural children were reported to engage in low levels of PA after school, on holidays, and on weekends, and this supports our findings that PA is significantly correlated with access to facilities and places for outdoor play and PA [37]. There are limited studies that compared the screen time exposure of preschool children between urban and rural regions. A few Western studies showed poor adherence to screen time guidelines among urban children [38,39], while another study showed mixed results [40]. A Japanese study revealed a low compliance to screen time guidelines among rural children [25]. A systematic review showed that rural environments hinder PA among children and adolescents, and instead, can facilitate screen-based sedentary behaviors, with such an influence more evident in preschool-aged children than in adolescents [41]. A study conducted on children living in rural regions in Japan also showed that perceived neighborhood factors, such as “sidewalks in neighborhood”, “paths for cycles”, and “aesthetic qualities” influenced outdoor exercise time and screen time [25]. These results are consistent with the findings of a Canadian study, where access to public transportation, parks and other green spaces, exercise facilities, and community recreation centers influenced PA [42]. The reasons for such phenomenon are that young children are highly dependent on their parents for activity, and if parents continue to limit outdoor playtime and instead promote activity inside the house or other places that are easily supervised by parents, children may increasingly become more dependent on TV or virtual games when seeking pleasure [43]. One effective means to address these problems and reduce screen time would be for parents to engage in outdoor activities with their children to provide experiences that are essential in early childhood, such as freedom of movement, fun, creativity, and confidence.

Second, parents’ DM variables that predicted MVPA among preschool children were parents’ DME and DMA in the urban regions and parents’ DME in the rural regions, and these remained significantly different between regions even after adjusting for sex, age, and BMI. Children’s PA and screen times are influenced by parents’ attitudes and beliefs, which can either hinder or facilitate children’s PA or screen times [44]. In one of our joint studies conducted in Finland, children’s PA and (digital media use) DMU were reported to be associated with parents’ participation, and the association strengthened with a decrease in the child’s age [24]. A literature review showed that parents’ PA and DME affect children’s health behaviors [21], and the importance of active parental participation increases further in the coming years with continued advances and access in digitalization. Although a growing number of parents value learning competencies over PA amidst the intensifying focus on academics even in early childhood, many studies continue to show that PA in early childhood has a strong impact on brain development [45]. Hence, parents’ active involvement in outdoor play or performing easy stretching exercises at home with their children can help to boost the practice of MVPA [26]. Indeed, our findings showed that parents’ DMA is associated with children’s MVPA. Previous studies also showed that people with poor health literacy are not likely to practice healthy behaviors, including PA [46], and are therefore at an increased health risk for disease [47]. A study on adolescents showed that moderate or high health literacy is positively associated with PA in leisure time [48]. However, unlike the cases of adults and adolescents, outdoor play and PA among preschool children is highly influenced by their parents’ awareness and positive actions for healthy health behaviors [49]. Therefore, parents’ function as good role models for their children as well as their health awareness positively contribute to children’s health behaviors [50].

Finally, this study showed that children’s screen time was significantly associated with parents’ DMH, DME, and DMA in the urban regions, and with parents’ DMH and DMA in the rural regions. Parents’ media-watching habits in leisure time were a common predictor of children’s screen times in both the urban and rural regions. Related Western research supports our findings [51,52], and a study on young children in Singapore-based research found a strong correlation of 0.9 or higher, between young children’s screen time and parental screen time, alluding to the close relationship between the two daily habits [53]. These results suggest that interventions that are focused on reducing screen time among preschool children should be based on family factors, such as parents’ involvement, and should start in early childhood, and target modifiable factors in the family environment [54]. Some research shows that increased screen time among children is linked to sex, age, the weekend, poorly educated parents, and high media access [55,56]. A Chinese study showed that children’s screen time declines with an increase in the health literacy of parents [57]. Even though preschool children are engaged in DM for a substantial duration, children with parents who are aware of DM guidelines showed relatively shorter use of DM, highlighting the importance of parents’ awareness of DM engagement guidelines for children. The associations between parents’ social factors (e.g., household income) and environmental factors, such as media access, play a pivotal role in evidence-based intervention studies that emphasize the need to limit prolonged screen time among children [58].

Our results must be interpreted with consideration of a few factors. First, the study data were collected from the reliable and validated SMALLQ^®^ using a culturally adapted version for Japan. The SMALLQ^®^ is a parent-report and recall questionnaire and thus may be vulnerable to self-report bias, social desirability bias, and recall bias [59]. Notwithstanding the limitations of subjective measures such as the questionnaire, mitigation strategies such as assurance of anonymity of survey responses and span of recall limited to the last 7 days were put in place to reduce biases. Second, the cross-sectional design of the study hinders making a causal inference between parents’ DMH, DME, and DMA and the adherence to the 24-h MG by region. Therefore, a longitudinal study or intervention study should be conducted to examine such relationships with greater clarity. Third, our study was conducted on preschool children in the Northeast and Kansai region of Japan, and therefore, the findings cannot be generalized to the entire preschool-age population in Japan. Fourth, in classifying urban and rural areas, the distinction was made based on the 1947 Japanese Enforcement Decree of Statutes of Local Governments, with the limitation that other demographic factors such as parental occupation, educational background, and household income could not be investigated. Our findings can bolster the evidence for various environmental factors involved in compliance with 24-h MG among preschool-aged children, and because these are strictly limited to preschool children in an Asian country, they present significant implications for developing effective interventions. In this study, we confirmed the relationship with parents’ DM to increase the 24-h MG compliance rate, but it is necessary to examine various factors applying the ecological model in a future study.

## 5. Conclusions

This research confirmed that parents’ DM is a strong predictor of adherence to 24-h MG among preschool children in Japan in urban and rural regions. Our findings showed that the 24-h MG (MVPA, screen time, sleep duration) of preschool children living in the urban and rural regions in Japan were different. Weekday MVPA was longer in the urban regions, while screen time on the weekends was longer in the rural regions. Further, this research showed that children’s screen time was significantly associated with parents’ DMH, DME, and DMA in the urban regions and with parents’ DMH and DMA in the rural regions. We specifically investigated parents’ DM engagement in relation to those of their children, which relatively has not attracted much research attention. Thus, our findings are significant for developing effective intervention programs for boosting compliance with 24-h MG that will contribute to ensuring the good health of preschool children.

## Figures and Tables

**Table 1:** Sociodemographic characteristics and urban/rural differences among parents and their children.

Variables	Total (*n* = 867)		Urban Region (*n* = 489)		Rural Region (*n* = 378)		*p*-Value
	*n* (%) or mean ± SD ^a^						
Characteristics of child							
Sex (girl: *n*, %)	410	(47.3)	235	(48.1)	175	(46.3)	0.607
Age (years: mean, SD)	4.6	±0.9	4.6	±0.9	4.5	±0.9	0.298
Height (cm: mean, SD)	106.9	±7.8	106.6	±7.7	107.4	±8.1	0.179
Weight (kg: mean, SD)	17.7	±3.0	17.4	±2.7	18.1	±3.4	<0.001
BMI (kg/m^2^: mean, SD)	15.4	±1.5	15.3	±1.4	15.6	±1.6	<0.001
BMI z-score (mean, SD)	−0.04	±0.84	−0.09	±0.83	0.05	±0.86	0.034
Characteristics of parents							
Sex (mother: *n*, %)	796	(91.8)	459	(93.9)	337	(89.2)	0.017
Age (years: mean, SD)	37.2	±5.1	38.1	±4.8	36.1	±5.2	<0.001
BMI (kg/m^2^: *n*, %)							
<18.49	102	(13.7)	64	(15.1)	38	(11.9)	<0.001
18.5–24.9	574	(77.4)	329	(77.8)	245	(76.8)	
>25.0	66	(8.9)	30	(7.1)	36	(11.3)	

^a^ SD, standard deviation. *p*-values were calculated using *t*-test for continuous variables and chi-square test for categorical variables.

**Table 2:** Digital media habits, engagement, awareness, and urban/rural differences among parents.

Variables	Total		Urban Region		Rural Region		*p*-Value
Parents’ digital media habits (min: mean, SD) ^a^							
Entertainment (weekday)	97.9	±79.5	98.7	±84.9	96.9	±72.1	0.747
Entertainment (weekend)	130.1	±104.0	116.0	±97.4	148.1	±109.3	<0.001
Entertainment (total)	113.1	±86.0	106.7	±86.3	121.1	±85.1	0.016
Social networking (weekday)	30.3	±41.2	29.8	±40.6	30.8	±42.1	0.741
Social networking (weekend)	34.3	±46.8	31.6	±44.7	37.8	±49.2	0.063
Social networking (total)	31.9	±42.1	30.4	±41.1	33.9	±43.5	0.246
Parents’ engagement with child (%: mean, SD) ^b^							
Physical play (weekday)	27.1	±28.4	28.5	±28.8	25.2	±27.8	0.101
Physical play (weekend)	46.7	±30.0	47.6	±30.9	45.5	±28.9	0.328
Physical play (total)	32.4	±26.5	33.8	±27.0	30.5	±25.8	0.069
Digital media (weekday)	46.8	±32.8	45.4	±32.8	48.7	±32.8	0.142
Digital media (weekend)	52.1	±31.2	50.2	±31.8	54.6	±30.2	0.041
Digital media (total)	48.2	±31.0	46.5	±31.1	50.4	±30.9	0.072
Parents’ digital media awareness (point: mean, SD) ^c^							
Limit digital media use	2.7	±1.2	2.7	±1.2	2.5	±1.2	0.085
Limit screen time to 1 h per day	2.7	±1.1	2.8	±1.1	2.6	±1.1	0.038
Introduce only educational programs	1.7	±1.0	1.8	±1.1	1.6	±1.0	0.030

^a^ SD, standard deviation. Group differences for continuous variables were assessed using *t*-tests. ^b^ Parents’ engagement with child. ^c^ Response option: (0) I am not aware, (1) I am not aware but practicing, (2) I am aware but do not practice, (3) I am aware and practicing.

**Table 3:** Urban/rural differences in children’s MVPA, screen time, sleep duration, and compliance with recommendations.

Variables	Total		Urban Region		Rural Region		*p*-Value
MVPA (min/day: mean, SD)							
Weekday	23.6	±35.4	26.8	±31.9	20.4	±38.3	0.017
Weekend	59.3	±65.9	59.4	±64.4	59.2	±67.7	0.958
Screen time (min/day: mean, SD)							
Weekday	87.4	±67.1	86.0	±67.0	89.3	±67.1	0.469
Weekend	126.4	±88.6	113.6	±80.2	143.7	±95.9	<0.001
Sleep duration (min/day: mean, SD)							
Weekday	606.7	±63.4	609.3	±60.2	603.0	±67.6	0.167
Weekend	634.5	±69.7	630.4	±68.5	639.8	±71.1	0.057
MVPA (% recommendations) ^a^							
Weekday	12.7		14.9		10.5		0.082
Weekend	35.8		34.1		37.6		0.314
Screen time (% recommendations) ^b^							
Weekday	23.0		24.6		21.0		0.209
Weekend	13.0		15.9		9.3		0.005
Sleep duration (% recommendations) ^c^							
Weekday	60.7		67.7		51.2		<0.001
Weekend	78.9		80.2		77.3		0.312

SD, standard deviation; MVPA, moderate-to-vigorous physical activity. Group differences for continuous variables were assessed using *t*-tests and for categorical variables were assessed using Wilcoxon signed-rank test. Additionally, ^a^ 180 min of total physical activity including 60 min/day of moderate-to-vigorous physical activity, ^b^ no more than 60 min/day for screen time, ^c^ between 10 and 13 h/day for sleep duration.

**Table 4:** Results from multiple regression analyses of 24-h movement guidelines and parents’ digital media habits, engagement, awareness for urban children.

Variables Associated to Parents	Urban Region ^a^											
	MVPA				Screen Time				Sleep Duration			
	b	(95% CI)	*β*	*p-*Value	b	(95% CI)	*β*	*p-*Value	b	(95% CI)	*β*	*p-*Value
Screen time on entertainment	−0.021	(−0.467, 0.024)	−0.049	0.360	0.340	(0.263, 0.415)	0.405	0.001	0.095	(−0.025, 0.166)	0.071	0.174
Screen time on social networking	0.035	(−0.054, 0.123)	0.042	0.183	−0.064	(−0.233, 0.105)	−0.039	0.455	−0.090	(−0.241, 0.060)	−0.060	0.240
Physical play engagement with child	0.604	(0.479, 0.729)	0.454	0.001	−0.374	(−0.636, −0.112)	−0.144	0.005	−0.128	(−0.352, 0.092)	−0.057	0.261
Digital media engagement with child	0.180	(0.061, 0.299)	0.158	0.003	0.061	(−0.160, 0.281)	−0.027	0.589	0.135	(−0.056, 0.326)	0.069	0.166
Limit digital media use for children	4.418	(1.337, 7.498)	0.148	0.005	−9.919	(−15.736, −4.103)	−0.167	0.001	1.293	(−3.818, 6.404)	0.025	0.619
Limit screen time to 1 h per day	4.436	(1.129, 7.744)	0.138	0.009	−15.636	(−21.634, −9.638)	−0.248	0.001	−0.303	(−5.622, 5.016)	−0.006	0.911
Introduce educational programs	1.283	(−2.103, 4.668)	0.040	0.457	−5.669	(−12.122, 0.784)	−0.088	0.085	4.271	(−1.219, 9.761)	−0.076	0.127

MVPA moderate to vigorous physical activity. b (95% CI), unstandardized coefficients and its 95% confidence interval. *β*, standardized coefficients. ^a^ Adjusted standardized regression coefficients for age, sex and BMI.

**Table 5:** Results from multiple regression analyses of 24-h movement guidelines and parents’ digital media habits, engagement, awareness for rural children.

Variables Associated to Parents	Rural Region ^a^											
	MVPA				Screen Time				Sleep Duration			
	b	(95% CI)	*β*	*p-*Value	b	(95% CI)	*β*	*p-*Value	b	(95% CI)	*β*	*p-*Value
Screen time for entertainment	0.024	(−0.026, 0.073)	−0.055	0.349	0.410	(0.317, 0.502)	0.452	0.001	0.036	(−0.56, 0.128)	0.045	0.443
Screen time for social networking	−0.061	(−0.152, 0.028)	−0.081	0.138	−0.082	(−0.278, 0.114)	−0.050	0.411	0.053	(−0.118, 0.223)	0.037	0.545
Physical play engagement with child	0.577	(0.450, 0.705)	0.472	0.001	−0.303	(−0.615, 0.010)	−0.113	0.057	0.194	(−0.127, 0.661)	0.169	0.154
Digital media engagement with child	0.146	(0.024, 0.267)	0.139	0.019	0.147	(−0.110, 0.403)	0.066	0.262	−0.046	(−0.273, 0.182)	−0.023	0.693
Limit digital media use for children	0.190	(−3.113, 3.492)	0.007	0.910	−1.780	(−8.803, 5.244)	−0.030	0.618	−0.794	(−7.120, 5.532)	−0.015	0.805
Limit screen time to 1 h per day	0.702	(−2.780, 4.184)	0.024	0.692	−5.877	(−13.198, 1.424)	−0.094	0.114	4.271	(−1.219, 9.761)	0.076	0.127
Introduce educational programs	3.307	(−0.578, 7.193)	0.102	0.095	−8.408	(−16.671, −0.145)	−0.122	0.046	2.007	(−5.415, 9.480)	0.032	0.597

MVPA moderate to vigorous physical activity. b (95% CI), unstandardized coefficients and its 95% confidence interval. *β*, standardized coefficients. ^a^ Adjusted standardized regression coefficients for age, sex and BMI.

## Data Availability

Data are contained within the article.
